# Comprehensive identification of contagious, environmental, and emerging microorganisms associated with bovine mastitis in Northern Minas Gerais, Brazil, using MALDI-TOF mass spectrometry

**DOI:** 10.14202/vetworld.2025.4196-4211

**Published:** 2025-12-31

**Authors:** Eliane Macedo Sobrinho Santos, Cintya Neves de Souza, Hércules Otacílio Santos, Livia Mara Vitorino da Silva, Geziella Aurea Aparecida Damasceno Souza, Leonardo Ferreira Oliveira, Maria Júlia Ribeiro Magalhães, Wagner Silva dos Santos, Agueda Maria de França Tavares, Renata Gabriela Chaves Ferreira, Anna Christina de Almeida

**Affiliations:** 1 Campus Araçuaí, Federal Institute of Northern Minas Gerais, Araçuaí, Minas Gerais, Brazil; 2 Institute of Agricultural Sciences, Federal University of Minas Gerais, Montes Claros, Minas Gerais, Brazil; 3 Department of Agricultural and Environmental Engineering, Federal University of Viçosa, Viçosa, Minas Gerais, Brazil

**Keywords:** MALDI-TOF MS, bovine mastitis, contagious pathogens, environmental pathogens, emerging microorganisms, microbial etiology, public health

## Abstract

**Background and Aim:**

Bovine mastitis remains one of the most economically significant diseases in dairy herds, driven by diverse etiological agents that vary in prevalence across regions and production systems. Rapid and reliable identification of mastitis-causing microorganisms is essential for targeted treatment, improved herd management, and enhanced biosecurity. This study aimed to identify and characterize the microorganisms associated with clinical and subclinical mastitis in dairy cows from northern Minas Gerais (Brazil) using Matrix-assisted laser desorption/ionization-time-of-flight mass spectrometry (MALDI-TOF MS), with special emphasis on uncommon and emerging bacterial species of potential public health concern.

**Materials and Methods:**

Milk samples (n = 321 isolates) were collected from cows diagnosed with clinical or subclinical mastitis between 2022 and 2024 across 15 farms. Bacteria were cultured on 5% sheep blood agar and identified by MALDI-TOF MS according to Bruker scoring criteria. Gram classification and contagious versus environmental categorization were performed. Descriptive statistics, chi-square analysis (p < 0.05), and Bray–Curtis similarity with Unweighted Pair Group Mathematical Average clustering were applied to determine distribution patterns and microbial diversity.

**Results:**

MALDI-TOF MS achieved an identification rate of 88%, predominantly at the species-level (99.38%). Gram-positive bacteria were significantly more frequent than Gram-negative bacteria (78%; χ² = 168.52; p < 0.000001). Most pathogens were classified as contagious (65%), followed by environmental agents (23%) (χ² = 64.40; p < 0.000001). The most prevalent organisms were Staphylococcus aureus (30.2%), Staphylococcus chromogenes (22.1%), and Sthaphylococcus epidermidis (4.9%). A combined frequency of 7.48% represented uncommon microorganisms, including Burkholderia cepacia, Arthrobacter koreensis, Ralstonia pickettii, Kosakonia radicincitans, Rothia terrae, and Paenibacillus azoreducens, some of which may pose emerging risks to bovine health and public health. Cluster analysis revealed two major microbial groups with distinct ecological and pathogenic profiles, highlighting the complexity of mastitis epidemiology in the region.

**Conclusion:**

This study provides an updated and region-specific overview of the mastitis microbiome in northern Minas Gerais, demonstrating the predominance of S. aureus and non-aureus staphylococci, alongside diverse environmental and rare pathogens. MALDI-TOF MS proved to be a powerful diagnostic tool for rapid species-level identification, supporting more precise mastitis control strategies. The detection of emerging or uncommon microorganisms underscores the need for sustained surveillance, improved biosecurity, and further research, including genomic characterization and antimicrobial resistance monitoring. These findings contribute to advancing dairy herd health, guiding targeted interventions, and informing One Health perspectives.

## INTRODUCTION

Bovine mastitis is one of the most prevalent diseases affecting dairy herds and continues to impose substantial economic losses on Brazilian dairy farming [1]. In Minas Gerais, the leading milk-producing state, the disease receives particular attention due to its recurrent impact on herd productivity and milk quality [2]. Studies conducted across different regions of Brazil report wide variability in subclinical mastitis prevalence, ranging from 16.1% to 81.9%, influenced by local environmental conditions, management practices, and milking hygiene [3–6]. Contagious pathogens such as *Staphylococcus aureus* and *Streptococcus agalactiae* remain among the most frequently detected agents in Minas Gerais, reflecting persistent challenges in milking routines and biosecurity practices [7, 8]. Economically, mastitis contributes to reduced milk yield and quality, increased culling and replacement of cows, and elevated expenditures on treatment and veterinary services [9]. While contagious mastitis involves direct cow-to-cow transmission, environmental mastitis arises when pathogens from bedding, soil, water, mud, or fecal contamination penetrate the mammary gland and induce inflammation [10].

A wide diversity of microorganisms, including bacteria, viruses, and fungi, has been associated with bovine mastitis worldwide [11–13]. Some uncommon microorganisms have also been reported; although rare, they may harbor virulence factors and antimicrobial resistance mechanisms that complicate clinical management [14]. Regardless of the type or frequency of pathogens involved, accurate identification remains essential to guide control strategies and improve treatment outcomes [15]. Matrix-assisted laser desorption/ionization-time-of-flight mass spectrometry (MALDI-TOF MS) has emerged as an efficient diagnostic tool for rapidly identifying a broad range of microorganisms with high accuracy. International studies demonstrate its effectiveness in detecting mastitis pathogens [16–18]. However, MALDI-TOF MS applications in Brazil remain limited, and the detection of uncommon mastitis-associated organisms requires broader, more systematic investigation.

Despite the recognized importance of bovine mastitis in Brazil and the extensive documentation of classical pathogens such as *Staphylococcus aureus* and *Streptococcus agalactiae* in Minas Gerais [7, 8], there remains a substantial gap in region-specific epidemiological information derived from advanced diagnostic technologies. Most studies rely on conventional microbiological or biochemical methods, which may fail to detect slow-growing, atypical, or emerging microorganisms capable of harboring virulence factors and antimicrobial resistance traits [11–13, 15]. Furthermore, the northern region of Minas Gerais, characterized by semi-arid conditions, distinct management systems, and environmental vulnerabilities, has been poorly represented in previous mastitis surveys, limiting the understanding of its unique microbial ecology. In addition, the presence and frequency of rare, opportunistic, or environmental pathogens remain largely undocumented due to the limited use of tools capable of species-level resolution, such as MALDI-TOF MS. This diagnostic gap restricts accurate assessment of microbial diversity, impedes early recognition of emerging threats, and undermines targeted mastitis control measures. Therefore, a comprehensive and updated characterization of contagious, environmental, and uncommon microorganisms using high-resolution analytical methods is urgently needed for this region.

To address these gaps, this study aimed to identify, classify, and characterize the microorganisms associated with clinical and subclinical bovine mastitis in dairy herds from northern Minas Gerais (Brazil) using MALDI-TOF mass spectrometry. Specifically, the study sought to (i) determine the relative frequency of Gram-positive, Gram-negative, contagious, and environmental pathogens circulating in the region; (ii) detect and document uncommon, opportunistic, and emerging microbial species relevant to bovine health and potential public health risks; and (iii) evaluate microbial diversity patterns using multivariate statistical approaches. By integrating advanced proteomic identification with epidemiological analysis, the study provides a region-specific diagnostic framework that supports more precise mastitis control strategies, enhances biosecurity, and contributes to broader One Health surveillance efforts.

## MATERIALS AND METHODS

### Ethical approval

All procedures involving animals in this study were performed in strict accordance with national and institutional guidelines for the ethical use of animals in research. Milk samples were collected exclusively from dairy cows on farms in northern Minas Gerais, Brazil, to determine the microbial etiology of clinical and subclinical mastitis. The study protocol, including sampling procedures, clinical examinations, and animal handling, was reviewed and approved by the Ethics Committee on Animal Use of the Federal University of Minas Gerais under protocol No. 90/2018.

Animal handling complied with the Brazilian National Council for the Control of Animal Experimentation regulations and adhered to guidelines established by the Brazilian College of Animal Experimentation. All clinical examinations were performed by trained professionals, minimizing distress and avoiding invasive procedures beyond routine diagnostic evaluations. Milk collection was conducted aseptically and without causing pain or harm to the animals. No animals were subjected to experimental induction of disease, and only naturally occurring cases of mastitis were included.

The study did not involve euthanasia, invasive sampling, or procedures that could compromise animal welfare. Farm owners provided informed consent prior to participation, and data confidentiality was maintained throughout the study. All efforts were made to safeguard animal health and well-being, ensuring full compliance with ethical, biosafety, and animal care standards.

### Study period and location

The study was conducted from January 2022 to December 2024 in the northern region of Minas Gerais, a semi-arid zone characterized by recurrent water scarcity due to irregular rainfall patterns [19]. Fifteen dairy farms were visited monthly during the study period as part of a longitudinal surveillance program. Sampling followed a self-generated nonprobabilistic approach aligned with the operational needs of partner farms. Cows were managed under extensive production systems with native pasture, grain silage, and commercial concentrate. Milking routines followed recommended hygienic practices, including pre-dipping and post-dipping procedures.

### Animal selection criteria

Animals were included in the study if they exhibited clinical signs of mastitis, such as udder swelling, redness, or clots in milk, or tested positive for subclinical mastitis using the California Mastitis Test (CMT ≥2). Cows that were undergoing antibiotic treatment or had recently completed treatment were excluded from sampling.

### Clinical evaluation and sample collection

Clinical mastitis was diagnosed using the cup test, whereas subclinical cases were identified through the CMT. A full clinical assessment was conducted, incorporating anamnesis, measurement of vital parameters (temperature, pulse, respiration), udder inspection and palpation, experimental milking, and organoleptic evaluation of milk to confirm mastitis.Aseptic techniques were used to collect milk samples from clinically and subclinically affected quarters. Samples were refrigerated immediately after collection and transported to the laboratory for analysis.

### Isolation and phenotypic characterization of bacteria

Milk samples were cultured on 5% (v/v) sheep blood agar using the depletion technique at the Animal Health Laboratory of the Federal University of Minas Gerais. Isolated colonies were examined for morphological characteristics, including pigment, colony size, and hemolysis patterns, and subsequently subjected to Gram staining for preliminary classification.

### Proteomic identification using MALDI-TOF mass spectrometry

A total of 321 bacterial isolates were identified at the AQUACEN/REN QUA Laboratory (Veterinary School, UFMG) using MALDI-TOF MS (Bruker Daltonics Microflex™, Germany). Isolates were plated on plate counting agar using the depletion technique and incubated aerobically at 37°C (±2) for 24–48 h. Following established protocols [20], colonies were transferred to a stainless-steel target plate, overlaid with 1 μL of 70% formic acid and 1 μL of α-cyano-4-hydroxycinnamic acid matrix. Instrument calibration was performed using a bacterial test standard (*Escherichia coli* DH5α, Bruker Daltonics).

Identification scores were interpreted according to the manufacturer’s criteria (Table 1). Each isolate was analyzed in duplicate, and identification was confirmed only when replicate scores were consistent. *E. coli* DH5α served as a positive control, while uninoculated medium was used as a negative control to ensure sterility and validate instrument performance.

**Table 1:** Matrix-assisted laser desorption/ionization-time-of-flight mass spectrometry real-time identification scoring criteria.

Score	Definition
2.30 a 3.00	highly probable identification of species
2.00 a 2.29	reliable identification of the genus and probable identification of the species
1.70 a 1.99	probable genus identification
0.00 a 1.69	unreliable identification

**Table 2:** Key characteristics of non-aureus *Staphylococcus*

Non-aureus *Staphylococcus*	Literature reports
*Staphylococcus haemolyticus*	It is considered an opportunistic environmental pathogen [32, 57]. However, it can colonize the skin and the apex of the teat, causing intramammary infection [37]. Freu *et al*. [32] and Jenkins *et al*. [58] reported that it was the second most frequently isolated *non-aureus Staphylococcus* species from mastitis cases.

*Staphylococcus hyicus*	This microorganism can be associated with different diseases, such as bovine mastitis and human sepsis, which makes it a threat to public health [59].

*Staphylococcus saprophyticus*	It is an important cause of urinary tract infections in humans and bovine mastitis [33].

*Mammaliicoccus sciuri*	It is environmental in nature [60]. Therefore, management practices can be a determining factor in infections caused by this bacterium. It is also a microorganism that carries a wide repertoire of antimicrobial resistance genes [34]. Studies have reported an increase in the frequency of this pathogen in bovine mastitis cases [32].

*Staphylococcus simulans*	It has been frequently observed in bovine mastitis cases [32, 37, 47]. The high frequency of this pathogen in previous studies can be attributed to its specificity for the udder, causing persistent intramammary infection [37, 47, 60–62].

*Staphylococcus warneri*	It is an opportunistic mastitis pathogen [63, 64] associated with the emergence of multidrug resistance characteristics among coagulase-negative *Staphylococcus* species, making it a public health concern [65]. The occurrence of this pathogen in cases of mastitis remains controversial [66–68].

*Staphylococcus xylosus*	It is a food-borne bacterium that is predominant among coagulase-negative *staphylococci* in cows affected by mastitis in various regions [69, 70]. Their ability to form biofilms can be a complicating factor for treating bovine mastitis in clinical practice [71, 72].

*Staphylococcus auricularis*	Other authors have also identified *S. auricularis* using MALDI-TOF analysis [73–76]. Gram-positive, aerobic, and non-sporulated cocci that can be part of the flora of the skin, ear, and mucous membranes.

### Statistical analysis

Descriptive and inferential statistics were applied to the microbiological data to determine frequency distributions and identification profiles. Graphs illustrating the occurrence of each microorganism detected by MALDI-TOF MS were generated using Microsoft Excel 2016 (Microsoft Corp., Washington, USA).

The distribution of Gram-positive versus Gram-negative organisms and the proportion of contagious versus environmental agents were evaluated using the chi-square test of adherence [21], with p < 0.05 considered statistically significant.

To examine species diversity, Bray–Curtis similarity coefficients were calculated[22], which are robust to compositional ecological data. Dendrogram reliability was assessed using Unweighted Pair Group Mathematical Average clustering with 1,000 bootstrap replicates, performed in Paleontological Statistics software (v2.17c). Descriptive statistics were completed in Microsoft Excel 2016, and multivariate analyses were conducted using PAST v2.17c [23].

## RESULTS AND DISCUSSION

### Identification performance of MALDI-TOF MS

This study presents important data on the etiological frequency of infectious bovine mastitis in northern Minas Gerais. Thirty-eight (11.84%) of the 321 samples analyzed by MALDI-TOF MS were not identified. The MALDI-TOF MS identification rate was 88%, 99.38% at the species-level and 0.62% at the genus level. Nonnemann *et al*. [24] obtained 93.5% and 6.5% identification at the species and genus levels, respectively, using the same technique.

**Figure 1:**
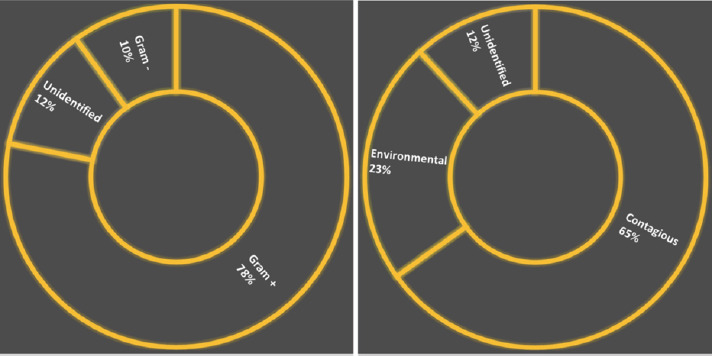
Distribution of Gram-positive and Gram-negative bacteria, as well as contagious and environmental micro-organisms, in cases of bovine mastitis on dairy farms in northern Minas Gerais, identified by Matrix-assisted laser desorption/ionization-time-of-flight mass spectrometry (n = 321).

Figure 1 shows that Gram-positive bacteria (78%) predominate in cases of mastitis, with Gram-negative bacteria accounting for 10%. Gram-positive bacteria were significantly more common (χ² = 168.52; p < 0.000001). The origin of the pathogen also varied, with 65% of isolates classified as contagious and 23% as environmental, as determined by MALDI-TOF MS (χ² = 64.40; p < 0.000001).

### Epidemiological classification of mastitis pathogens

Characterization of mastitis pathogens is essential for optimizing control and treatment strategies. Although some microorganisms have contagious and environmental characteristics [25, 26], contagious pathogens usually cause subclinical mastitis and spread in the mammary gland among cows. Environmental pathogens cause clinical mastitis by infecting the udder through exposure to feces, mud, soil, and bedding [27].

Although some microorganisms have mixed characteristics, they are classified as either environmental or contagious. However, the habitat and transmission of certain agents remain a topic of debate in the literature. For example, *Klebsiella* and *Streptococcus uberis* are generally environmental, but persistent infections can act as contagious agents [28, 29].

The classification of *Staphylococcus non-aureus* (formerly known as coagulase-negative *Staphylococcus*) is particularly debated [30–32]. Many of these pathogens act as opportunistic and secondary pathogens that colonize the skin and root canal [10]. Species such as *Staphylococcus saprophyticus*, *Staphylococcus sciuri* (now *Mammaliicoccus sciuri*), and *Staphylococcus simulans* are found in the environment. In addition to causing outbreaks, they can spread antibiotic resistance and act as cross-infections between humans and animals, raising public health concerns [32–34]. The epidemiology of non-aureus *Staphylococcus* in clinical and subclinical mastitis remains poorly understood. Therefore, this study grouped them with *S. aureus*.

### MALDI-TOF MS score interpretation and diagnostic reliability

**Figure 2:**
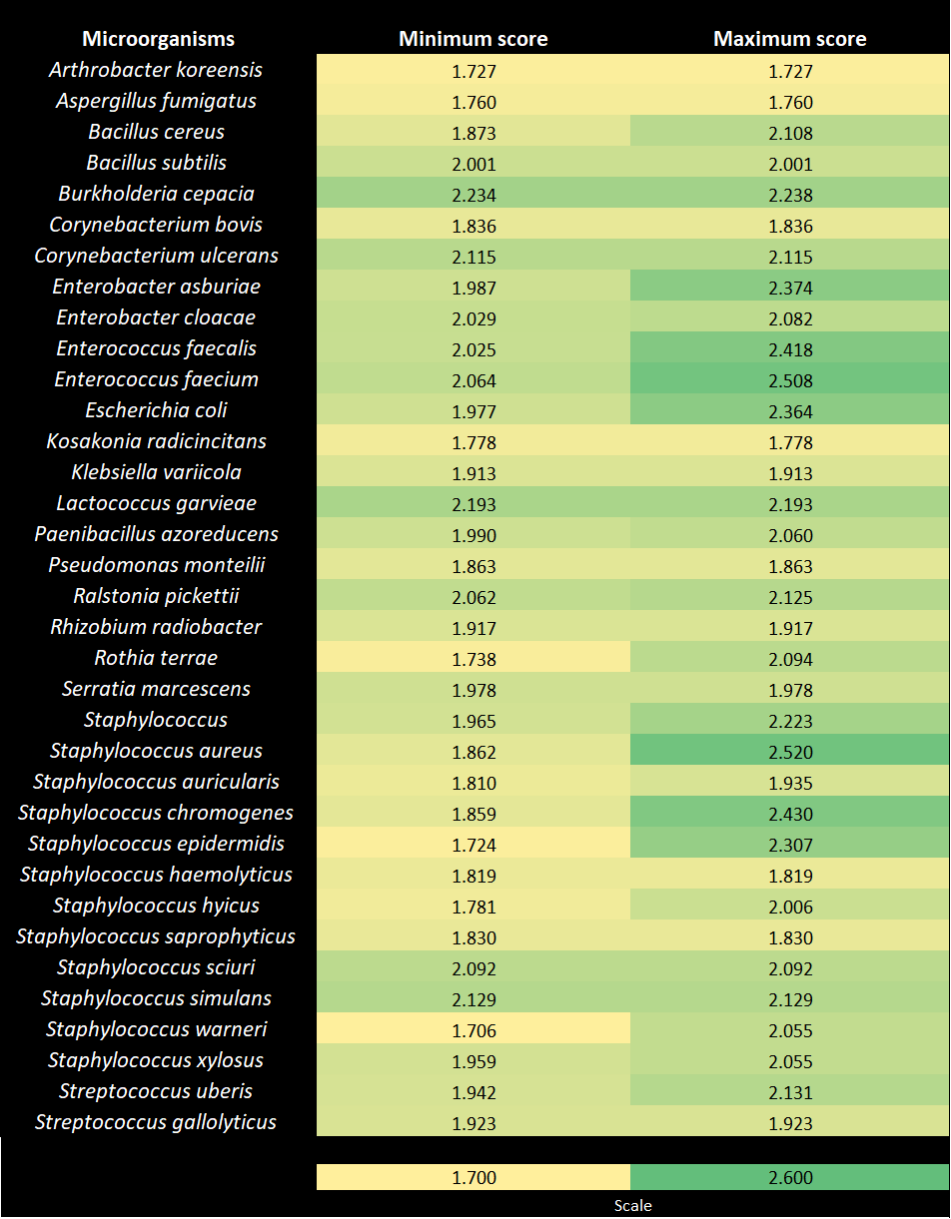
Heat map showing identification scores for environmental and contagious microorganisms identified by Matrix-assisted laser desorption/ionization-time-of-flight mass spectrometry.

The environmental and contagious microorganisms identified by MALDI-TOF MS are shown in Figure 2. The assigned scores reflect the level of confidence in species and genus identification. All isolates were reliably identified at least at the genus level. Most specimens showed high-confidence identification at the species-level.

### Frequency distribution of identified microorganisms

**Figure 3:**
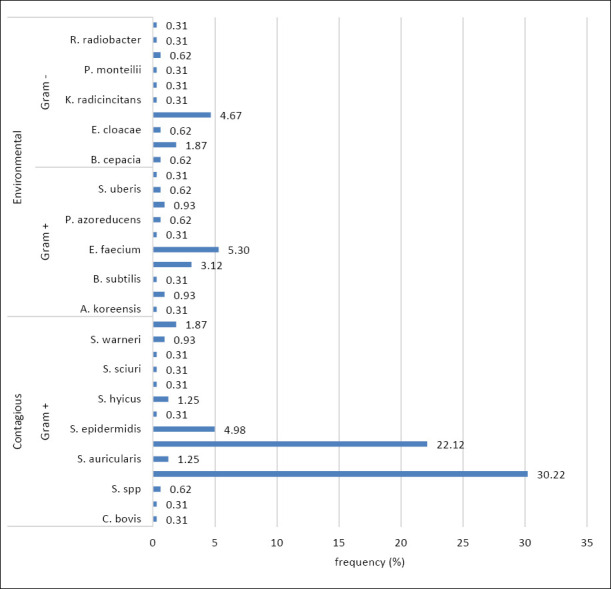
Distribution of environmental and contagious microorganisms in cases of bovine mastitis on dairy farms in northern Minas Gerais, classified as Gram-positive or Gram-negative and identified by Matrix-assisted laser desorption/ionization-time-of-flight mass spectrometry (n = 321).

Figure 3 shows the frequency of the microorganisms. According to the literature, contagious pathogens were predominant, associated with agents such as *Streptococcus agalactiae*, *Staphylococcus* spp., *Corynebacterium* spp., and *Mycoplasma* spp., which cause subclinical and persistent infections [35]. In this study, the most common contagious strains were *S. aureus* (30.2%), *Staphylococcus chromogenes* (22.1%), and *Staphylococcus epidermidis* (4.9%). Other *Staphylococcus* species, such as *Staphylococcus haemolyticus*, *Staphylococcus hyicus*, *S. saprophyticus*, *S. sciuri*, *S. simulans*, *Staphylococcus warneri*, *Staphylococcus xylosus*, and *Staphylococcus auriculares*, accounted for 7.1% of the total. The frequency of non-aureus *Staphylococcus* varies across the studies [30, 32, 36–38]. *Corynebacterium* represented 0.62% of the isolated strains. *Enterococcus* spp. (8.4%), *E. coli* (4.7%), and *Enterobacter* spp. (2.5%) were also identified. Other microorganisms, including *Klebsiella variicola*, *Lactococcus garvieae*, *Pseudomonas monteilii*, *Rhizobium radiobacter*, *Aspergillus fumigatus*, *Bacillus cereus*, *Bacillus subtilis*, *Streptococcus uberis*, *Streptococcus gallolyticus*, and *Serratia marcescens*, had a combined frequency of 4.09%. Nonneman *et al*. [24] identified 24 genera and 61 species, including *Staphylococcus*, *Streptococcus*, enterobacteria, and coryneform bacteria.

Environmental pathogens such as *E. coli*, *Klebsiella pneumoniae*, *Enterobacter* spp., *Proteus* spp., *Serratia* spp., *Pseudomonas aeruginosa*, *Streptococcus*, fungi, and algae are opportunistic [35, 39]. They cause transient clinical mastitis, which is often associated with severe cases [15, 17, 35].

### Detection of uncommon and low-frequency pathogens

Uncommon bacteria, including *Arthrobacter koreensis*, *Burkholderia cepacia*, *Kosakonia radicincitans*, *Paenibacillus azoreducens*, *Ralstonia pickettii*, and *Rothia terrae*, were recorded with a frequency of 3.39% (Figure 3). The occurrence of these bacteria warrants discussion, as they contribute to mastitis and may pose a risk to public health. The low-frequency (below 1%) of certain microorganisms in known habitats, such as soil or water, may indicate contamination during sample collection or processing. Such agents can be seen as indicators of hygiene failures, reinforcing the need for training and better protocols, especially during milking.

### Microbial grouping based on Bray–Curtis similarity

**Figure 4:**
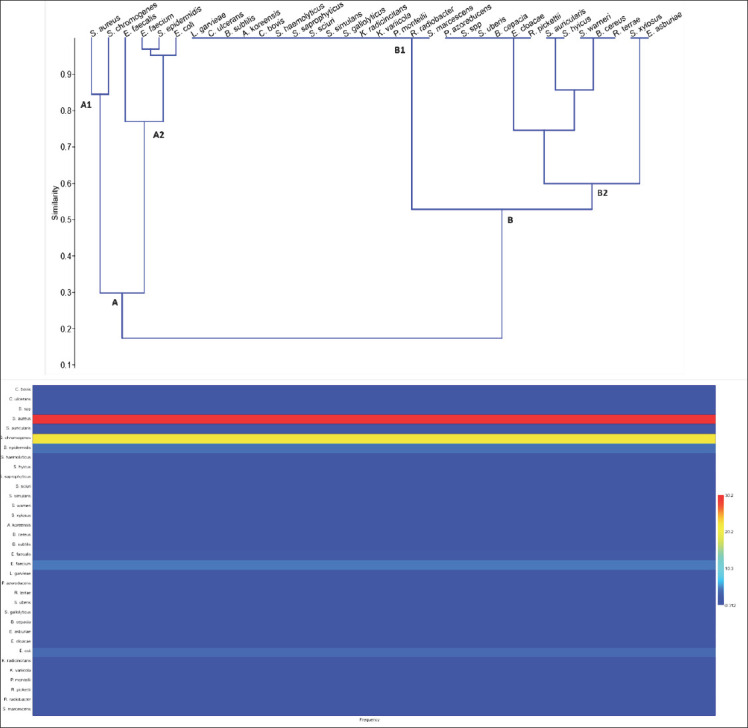
Bray–Curtis similarity dendrogram prepared using the Unweighted Pair Group Mathematical Average for the frequencies of microorganisms in cases of bovine mastitis on a dairy farm in northern Minas Gerais.

The dendrogram (Figure 4) revealed two microbial groups (A and B) with frequency levels below 20%. Group A, more closely linked to mastitis, included *S. aureus*, *S. chromogenes*, *Enterococcus faecalis*, *Enterococcus faecium*, *S. epidermidis*, and *E. coli*. This group was divided into two subgroups (A1 and A2), with 30% similarity between them. Microorganisms within each subgroup had more than 75% similarity in frequency.

### Group A: Major mastitis-associated cluster

#### Subgroup A1: Dominant contagious agents

A1 consists of *S. aureus* and *S. chromogenes*, both of which are contagious. Most *S. aureus* strains were identified by MALDI-TOF MS with a score of >2.0 (Figure 2), indicating probable species identification. *S. aureus* is one of the main agents of contagious mastitis and a risk to consumers, as it can produce thermostable enterotoxins that resist milk processing [40]. MALDI-TOF is a reliable tool for identifying subclinical mastitis at the species-level in cows [41–43]. *Staphylococcus chromogenes* has the highest incidence of bovine mastitis in dairy herds. It is associated with persistent infections, high somatic cell count, biofilm production, and plasma coagulation in some isolates [44].

#### Subgroup A2: Mixed environmental–contagious profile

A2 includes *S. epidermidis* (classified as contagious) along with environmental bacteria, such as *E. faecalis*, *E. faecium*, and *E. coli*. *S. epidermidis* is often detected in milk and negatively impacts the quality and quantity of the product. Its presence in clinical and subclinical mastitis is associated with poor hygiene or hand contamination [45]. It was the third-most-isolated microorganism in this study. Previous research indicates it is the most prevalent non-aureus Staphylococcus and is more commonly associated with subclinical mastitis than with clinical mastitis.

*E. coli*, *Enterobacter*, and *Enterococcus* bacteria are linked to environmental mastitis etiology. The identification scores for E. coli and Enterococcus by MALDI-TOF MS were consistently above 2 (Figure 2), confirming the identification accuracy. The scores for *Enterobacter* and *Enterococcus* ranged from 1.987 to 2.374. *E. coli* mastitis can be acute or hyperacute, occurring during lactation and drying off, and can lead to animal death despite control measures [49]. Like *E. coli*, *Enterococcus* and *Enterobacter* are inhabitants of the gastrointestinal tract of warm-blooded animals and are present in soil and water [50]. Other studies have confirmed the efficiency of MALDI-TOF in identifying *E. coli* [24], *Enterococcus* [51], and *Enterobacter* [52, 53].

### Group B: Less frequent and highly diverse microorganisms

Group B includes the remaining microorganisms, which are divided into subgroups B1 and B2 (Figure 4). Both contain contagious and environmental species, with identification scores shown in Figure 2. Subgroup B1 (100% similarity) contains the least frequent microorganisms, including contagious species (*Corynebacterium bovis, Corynebacterium ulcerans, S. haemolyticus, S. saprophyticus, S. sciuri, S. simulans*, and *S. gallolyticus*) and environmental species (*A. koreensis, B. subtilis, K. radicincitans, K. variicola, L. garvieae, P. monteilii, R. radiobacter*, and *S. marcescens*). Many of these species have already been reported as mastitis agents [32–34, 36, 37, 47]. *Corynebacterium bovis* is associated with subclinical mastitis due to intramammary infection [54]. Some non-aureus *Staphylococcus* species (*S. chromogenes, S. epidermidis, S. saprophyticus, S. sciuri, S. simulans, S. warneri*, and *S. xylosus*) are relevant because they can produce bacteriocins and inhibit *S. aureus* growth [55, 56]. Although some non-aureus *Staphylococcus* species are rare on dairy farms in northern Minas Gerais, the literature highlights their relevance [32–34, 37, 47, 57–76].

### Detailed notes on rare and emerging pathogens

No reports associated *A. koreensis* with cases of clinical or subclinical mastitis. This bacterium was detected by MALDI-TOF MS in isolates from northern Minas Gerais with a frequency of 0.31% (Figure 3). *A. koreensis* is tolerant to desiccation and is isolated from the rhizosphere of *Nerium oleander*. It promotes plant growth [77]. Its occurrence may be linked to the use of bioinputs. Further studies are required to investigate this fact.

*Kosakonia radicincitans* (formerly *Enterobacter*) has been isolated from plants, with some strains considered facultative human pathogens [78]. No evidence of mastitis has been found. Reports in the literature are scarce, perhaps because it lacks superior pathogenic or resistance properties compared to Enterobacter [79]. Although *K. radicincitans* is known to improve plant performance [80, 81], there are rare reports of human infections. Therefore, it should not be ignored in the context of One Health. Although caution is required in interpreting its occurrence in 0.31% (Figure 3), it may be a potentially significant agent with a MALDI-TOF MS score of 1.778. Further studies on cases of mastitis are needed.

*Klebsiella variicola* is associated with severe mastitis, which can lead to death or reduced milk production [28]. There are few studies on *L. garvieae* in bovine mastitis. However, it is an emerging zoonotic pathogen with the potential to cause disease [82]. *Bacillus subtilis* has potential as a probiotic and may help prevent mastitis in dairy cows [83].

The occurrence of *S. marcescens* was 0.31% (Figure 3) on farms in northern Minas Gerais. This finding is important because it identifies an emerging bacterium resistant to multiple antimicrobials [84, 85]. Studies in China in 2016 and 2023 showed this pathogen in 1.1% and 1.5% of mastitis samples, respectively [85, 86]. Higher rates were found in Korea (4.5%) and Finland (35%–39%) [87].

Subgroup B2 comprises 13 microorganisms, including the fungus *A. fumigatus*, which can cause serious infectious diseases in animals, including mastitis, and can penetrate and contaminate the mammary gland [88].

Uncommon microorganisms should not be overlooked. They can carry virulence genes, develop antibiotic resistance, and pose public health risks. *B. cepacia* (formerly *Pseudomonas cepacia*) had a frequency of 0.62%, with a MALDI-TOF MS detection score of >2.2 (Figure 2). This bacterium is found in river sediments and in plant rhizosphere soils [89, 90]. It is considered an adaptable and opportunistic pathogen that poses a life-threatening risk to humans and animals [89, 91]. This bacterium has been detected in cases of ovine mastitis [92] and in cows with severe mastitis (grade 3) kept in a compost barn system [8]. These findings require further investigation of the mechanism of action of *B. cepacia* in bovine mastitis. In any case, the authors recommend caution in the use of bacterial biopesticides and bioremediations based on the *B. cepacia* complex.

The *P. azoreducens* species was detected by MALDI-TOF MS with a frequency of 0.62% and a score of 2 (Figure 2). This pathogen can be isolated from different environments, such as animal waste, plant parts, and milk [93–95] and produces antimicrobial compounds that are of interest to researchers in health and agricultural sciences [96, 97]. Due to its antimicrobial potential, this species has become a target for the food and pharmaceutical industries [96, 98]. These characteristics indicate that it may be an important bacterium in cases of persistent bovine mastitis. Therefore, further studies are needed to elucidate its role in the pathogenesis of infectious bovine mastitis.

*Ralstonia pickettii* was detected at a frequency of 0.62% in the evaluated mastitis cases. The confidence index in MALDI-TOF MS ranged from 2.06 to 2.12 (Figure 2). Evidence of an association between this bacterium and cases of mastitis is rare. A study that evaluated the differences between the microbiomes of healthy mice and those induced with clinical mastitis found the presence of *R. pickettii* among the differential microorganisms [99].

Among the microorganisms uncommon in bovine mastitis, *R. terrae* (ter′rae. L. gen. n. terrae, from the earth, referring to the organism isolated from the soil) [100] was the most frequent bacterium on dairy farms in northern Minas Gerais, with a rate of 0.93% and a MALDI-TOF MS detection confidence score ranging from 1.74 to 2.09 (Figure 2). Although rare, this bacterium has been reported in the scientific literature to be involved in bovine mastitis [101, 102], highlighting its importance from a public health perspective.

Mastitis caused by *B. cereus* often results in severe tissue damage and can lead to the production of abnormal mammary secretions [103]. *Streptococcus uberis* is an environmental pathogen that can cause chronic infection of the mammary gland, leading to both clinical and subclinical mastitis [104].

Studies showing the occurrence of other microorganisms, such as *R. radiobacter, P. monteilii, C. ulcerans*, and *S. gallolyticus*, also detected at the same frequency (0.31%) in cases of bovine mastitis in northern Minas Gerais, are scarce (Figure 3).

### Implications for mastitis control and diagnostic advances

The presence of various environmental and contagious microorganisms on farms in northern Minas Gerais highlights the need for effective mastitis management practices. To ensure effective prevention and treatment, control strategies must be specific to the nature of mastitis (contagious or environmental) [25, 40, 44]. Control is more complex for microorganisms with mixed characteristics or uncertain epidemiology.

The identification of microorganisms by MALDI-TOF MS is fundamental to the advancement of health practices in infectious mastitis. The Spectra Veterinary Database is constantly being updated. This may lead to the emergence of new species or to the expansion of the Spectra library. With routine MALDI-TOF MS diagnosis in several countries, knowledge of infections caused by *Staphylococcus* non-aureus and other uncommon microorganisms in bovine mastitis is expected to increase.

## CONCLUSION

This study provides a comprehensive characterization of the etiological agents associated with clinical and subclinical bovine mastitis in northern Minas Gerais, revealing important epidemiological insights for the region. Using MALDI-TOF MS, 88% of the 321 isolates were successfully identified, with 99.38% classified at the species-level. The predominance of Gram-positive organisms (78%) and the high occurrence of contagious pathogens, particularly *S. aureus* (30.2%) and *S. chromogenes* (22.1%), underscore the critical role of cow-to-cow transmission in mastitis dynamics. Environmental pathogens such as *E. coli, Enterococcus* spp., and *Enterobacter* spp. were also detected, further complicating mastitis etiology. The study further documents a diverse range of uncommon microorganisms, including *A. koreensis, B. cepacia, K. radicincitans, P. azoreducens, R. pickettii*, and *R. terrae*, highlighting both emerging pathogens and potential indicators of hygiene lapses. Bray–Curtis clustering revealed two distinct microbial groups, with *S. aureus, S. chromogenes*, and *S. epidermidis* demonstrating strong associations with mastitis cases.

Practical implications of these findings are significant for dairy herd management. The coexistence of contagious, environmental, opportunistic, and emerging pathogens reinforces the need for integrated mastitis control programs that combine rigorous milking hygiene, targeted treatment protocols, environmental sanitation, and routine microbiological monitoring. The identification of rare microorganisms further underscores the importance of improved sampling practices, aseptic milking techniques, and training farm personnel to reduce contamination risks. Additionally, the confirmed reliability of MALDI-TOF MS supports its use as a rapid, cost-effective diagnostic tool for herd-level surveillance and pathogen-specific interventions.

Among the strengths of this study are the longitudinal sampling across multiple farms, the use of a high-resolution proteomic identification method (MALDI-TOF MS), and the detailed ecological analysis of pathogen diversity using Bray–Curtis similarity. The detection of uncommon microorganisms adds novel contributions to the epidemiological understanding of mastitis in semi-arid Brazilian dairy systems.

However, limitations should be acknowledged. Some isolates (12%) could not be identified, likely due to gaps in the MALDI-TOF reference database or sample quality. The presence of low-frequency microorganisms may reflect environmental contamination or incidental occurrence, requiring cautious interpretation. Additionally, the study did not include antimicrobial susceptibility testing, which is essential for guiding therapeutic decisions.

The future scope of research should focus on expanding MALDI-TOF MS spectral libraries for veterinary pathogens, integrating genomic and antimicrobial resistance profiling, and conducting risk-factor analyses to correlate pathogen occurrence with farm management, climate, and hygiene conditions. Studies examining the pathogenicity of rare organisms such as *A. koreensis, K. radicincitans*, and *R. terrae* are also needed, given their potential One Health relevance.

In conclusion, the study emphasizes the multifactorial nature of bovine mastitis in northern Minas Gerais and demonstrates the value of MALDI-TOF MS in enhancing diagnostic precision, identifying emerging pathogens, and informing evidence-based control strategies. Strengthening mastitis monitoring programs, improving farm hygiene practices, and investing in advanced diagnostic tools will be essential for reducing disease burden, improving milk quality, and safeguarding animal and public health.

## DATA AVAILABILITY

All the generated data are included in the manuscript.

## AUTHORS’ CONTRIBUTIONS

ACDA, EMSS, and CNDS: Study design and conception. EMSS, CNDS, and HOS: Data analysis and drafted the manuscript. LFO, MJRM, and WSS: Statistical analysis, results interpretation, and literature review and drafted and revised the manuscript. AMFT, RGCF, LMVS, and GAADS: Sample collection and data collection and analysis. All authors have read and approved the final version of the manuscript.
